# 6-Mercaptopurine reduces cytokine and Muc5ac expression involving inhibition of NFκB activation in airway epithelial cells

**DOI:** 10.1186/s12931-015-0236-0

**Published:** 2015-06-19

**Authors:** Kondababu Kurakula, Anouk A. Hamers, Pieter van Loenen, Carlie J.M. de Vries

**Affiliations:** Department of Medical Biochemistry, Academic Medical Center, University of Amsterdam, Meibergdreef 15, 1105 AZ Amsterdam, The Netherlands; Present address: Department of Molecular Cell Biology, Leiden University Medical Center, Leiden, The Netherlands

**Keywords:** 6-MP, Epithelial cells, TNFα, Muc5ac, NFκB

## Abstract

**Background:**

Mucus hypersecretion and excessive cytokine synthesis is associated with many of the pathologic features of chronic airway diseases such as asthma. 6-Mercaptopurine (6-MP) is an immunosuppressive drug that is widely used in several inflammatory disorders. Although 6-MP has been used to treat asthma, its function and mechanism of action in airway epithelial cells is unknown.

**Methods:**

Confluent NCI-H292 and MLE-12 epithelial cells were pretreated with 6-MP followed by stimulation with TNFα or PMA. mRNA levels of cytokines and mucins were measured by RT-PCR. Western blot analysis was performed to assess the phosphorylation of IκBα and luciferase assays were performed using an NFκB reporter plasmid to determine NFκB activity. Periodic Acid Schiff staining was used to assess the production of mucus.

**Results:**

6-MP displayed no effect on cell viability up to a concentration of 15 μM. RT-PCR analysis showed that 6-MP significantly reduces TNFα- and PMA-induced expression of several proinflammatory cytokines in NCI-H292 and MLE-12 cells. Consistent with this, we demonstrated that 6-MP strongly inhibits TNFα-induced phosphorylation of IκBα and thus attenuates NFκB luciferase reporter activity. In addition, 6-MP decreases Rac1 activity in MLE-12 cells. 6-MP down-regulates gene expression of the mucin Muc5ac, but not Muc2, through inhibition of activation of the NFκB pathway. Furthermore, PMA- and TNFα-induced mucus production, as visualized by Periodic Acid Schiff (PAS) staining, is decreased by 6-MP.

**Conclusions:**

Our data demonstrate that 6-MP inhibits Muc5ac gene expression and mucus production in airway epithelial cells through inhibition of the NFκB pathway, and 6-MP may represent a novel therapeutic target for mucus hypersecretion in airway diseases.

**Electronic supplementary material:**

The online version of this article (doi:10.1186/s12931-015-0236-0) contains supplementary material, which is available to authorized users.

## Background

Chronic airway diseases such as asthma, chronic bronchitis, cystic fibrosis, and chronic allergic rhinitis are characterized by airway inflammation and mucus hypersecretion. Airway mucus is a component of the pulmonary innate immune function and plays a crucial role in defense against infectious and environmental agents [1–7]. Excessive mucus production is a hallmark in the pathogenesis of several airway diseases as it increases morbidity and mortality by obstructing mucocilary clearance and air flow [8]. Goblet cells produce mucins, a class of mucus glycoproteins that provide airway with characteristic adhesiveness and viscoelasticity to maintain epithelium homeostasis [3]. To date, more than 20 mucin genes have been identified in airways. Among several mucin genes, Muc5ac is a major constituent of the mucous layer in the airways of humans with respiratory diseases and therefore serves as a marker for mucus cell hyperplasia [9, 10]. Numerous previous studies reported that inflammatory cytokines, like TNFα, induce Muc5ac gene expression through activation of the NFκB pathway in lung epithelial cells [11]. NFκB is a major transcription factor essential for regulation of both innate and adaptive immunity, and inflammation. Inhibition of the NFκB pathway resulted in attenuation of airway inflammation in asthma both in experimental models and in humans [12–14]. A putative NFκB site at −3594/−3581 was identified in the promoter region of Muc5ac which is responsible for the increased Muc5ac expression following stimulation with inflammatory cytokines in airway epithelial cells [11].

Azathioprine is an immunosuppressive drug, which has been used for more than five decades to treat many inflammatory diseases [15–17]. Azathioprine is a pro-drug that is rapidly converted to 6-mercaptopurine (6-MP). As an immunosuppressive drug, 6-MP is widely used as a key agent in organ transplant recipients to prevent allograft rejection, as a maintenance drug for patients with inflammatory bowel disease, to treat rheumatoid arthritis, hematologic malignancies, chronic active hepatitis, and lupus nephritis [17]. 6-MP has been shown to have anti-inflammatory effects through inhibiting prostaglandin synthesis and neutrophil trafficking into inflammatory tissue [18] and Rac1 inhibition in T cells and gut epithelial cells [19, 20]. Several randomized trials reported that 6-MP led to improvement in patient’s asthmatic symptoms, probably due to reducing airway inflammation [18]. It is also reported that 6-MP may be used as a steroid sparing agent for patients with asthma [18]. Prolonged treatment of 6-MP has also been shown to be effective in the treatment of chronic asthma patients [17]. 6-MP may reduce T-cell activation and regulate the T-helper (Th)1 response to maintain a balance between Th1 and Th2 response in asthma [21]. Despite its extensive use in many clinical studies in asthma, the molecular mechanism behind actions of 6-MP is poorly understood.

Given that 6-MP has both an anti-inflammatory function and an immune modulatory function, and also its association with treatment of chronic asthma in humans, we hypothesized that 6-MP may suppress mucus production through inhibition of the NFκB pathway in airway epithelial cells. Our results clearly demonstrate that 6-MP strongly inhibits cytokine synthesis and mucus production by reduced gene expression of Muc5ac through suppression of the NFκB pathway in airway epithelial cells.

## Methods

### Cell culture and transfection

Human mucoepidermoid carcinoma (NCI-H292) cells were grown and maintained in RPMI 1640 medium (Life Technologies) containing 10 % FCS and 1 % penicillin/streptomycin. MLE-12 cells were grown in DMEM medium (Life Technologies) containing 5 % FCS and 1 % penicillin/streptomycin. For transient transfection experiments, cells were seeded at density of 2.4 × 10^4^ cells/ml and were transfected with indicated plasmids using Lipofectamine LTX plus transfection reagent (Life Technologies) according to manufacturer’s instructions.

### MTT assay

Cell viability was assessed by the MTT (3-[4, 5-dimethylthiazol-2-yl]-2, 5-diphenyltetrazolium bromide) (Sigma) assay. Cells were seeded in a 96-well plate at a density of 2.4 × 10^3^ cells/well and incubated overnight. Cells were made quiescent by incubation in medium without FCS for 24 h and then treated with vehicle (DMSO) or various concentrations of 6-MP (Sigma; 6-MP was dissolved in DMSO at a concentration of 10 mM) overnight followed by FCS (10 % v/v) stimulation for 24 h. After the incubation, cells were incubated with 10 μL of MTT reagent (5 mg/ml) for 3 h at 37 °C. The MTT reagent was removed, 100 μL of isopropanol was added to each well and incubated for 15 min. Colorimetric analysis was performed with an ELISA plate reader. Each experiment (in quadruplicate) was repeated at least three times.

### Semi-quantitative RT-PCR

Semi-quantitative RT-PCR (RT-PCR) was performed as described previously [22, 23]. Briefly, cells were serum-starved for 24 h and were pre-treated overnight with 6-MP (10 μM) or BAY-117085 (NFkB inhibitor; 10 μM: Calbiochem). After the incubation, cells were stimulated with TNFα (R&D systems; 50 ng/ml) or PMA (Sigma; 1nM) for 6 h before harvesting. Acidic ribosomal phosphoprotein P0 was used as a house-keeping gene. The analysis of the data involved the so called LinReg method described previously [24]. The following primers were used for RT-PCR: RANTES forw: 5′-CGCTGTCATCCTCATTGC-3′, RANTES rev: 5′-CCACTGGTGTAGAAATACTCC-3′; IL-6 forw: 5′-CGCCTTCGGTCCAGTTG-3′, IL-6 rev: 5′- TCGTTCTGAAGAGGTGAGTG-3′; IL-12p35 forw: 5′- AGGAATGTTCCCATGCCTTCAC-3′, IL-12 p35 rev: 5′- GCAACTCTCATTCTTGGTTAATTC-3′; TNFα forw: 5′-AGGACACCATGAGCACTGAAAG-3′, TNFα rev: 5′-AGGAGAGGCTGAGGAACAAG-3′; IL-1β forw: 5′-TGGCAGAAAGGGAACAGAAAGG-3′, IL-1β rev: 5′- GTGAGTAGGAGAGGTGAGAGAGG-3′; Muc5ac forw: 5′-GGAACTGTGGGGACAGCTCTT-3′, Muc5ac rev: 5′-GTCACATTCCTCAGCGAGGTC-3′; Muc2 forw: 5′-CAGCACCGATTGCTGAGTTG-3′, Muc2 rev: 5′- GCTGGTCATCTCAATGGCAG-3′; P0 forw: 5′-TCGACAATGGCAGCATCTAC-3′, P0 rev: 5′-ATCCGTCTCCACAGACAAGG-3′.

### Western blot analysis

Western blot analysis was performed as described previously [22, 23]. Antibodies applied in western blot analysis were phospho-specific (p)IkBα (Cell signaling; 1:500), and β-actin (Cell signaling; 1:1000).

### Periodic acid schiff staining

To evaluate the production of mucus glycoproteins, Periodic Acid Schiff (PAS) staining was performed. Cells were serum-starved for 24 h and then stimulated with TNFα (50 ng/ml) or PMA (1nM) for 24 h. After the incubation, cells were fixed with formaldehyde for 30 min and mucus glycoconjugates were visualized by PAS staining. Hematoxylin staining was also incorporated as a counterstain.

### Luciferase assays

Cells were transiently transfected with a NFκB reporter plasmid and a NFκB subunit p65 reporter plasmid using Lipofectamine LTX plus transfection reagent according to the manufacturer’s protocol and assay was described previously [25]. The construct containing the NFκB response element of the minimal IL-6 promoter was kindly provided by Dr. Karolien De Bosscher (Ghent University, Belgium) and was described previously [26]. The pRL-TK Renilla reporter plasmid (Promega) was co-transfected as an internal control for transfection efficiency. Luciferase activity measurements were performed using the dual-luciferase reporter assay system (Promega) and Glomax multi detection system (Promega) according to the manufacturer’s protocol. Each experiment (in duplicate) was repeated at least three times.

### Immunofluorescence staining

Cells were seeded on cover slips and serum-starved for 24 h. After starvation, cells were treated with 6-MP overnight and then stimulated with TNFα (50 ng/ml) for 24 h. Cells were fixed with 4 % (w/v) Formal-Fix (Thermo Scientific), washed and incubated with Muc5ac antibody (Santa Cruz; 1:500). Following repeated washing steps with PBS, protein localization was visualized by secondary antibodies coupled to fluorescent dyes Alexa Fluor-568 or −488 (Molecular Probes). Nuclei were counterstained with Hoechst (Molecular Probes).

### Statistical analysis

All statistical analyses were carried out with GraphPad Prism software (GraphPad Software, San Diego, Calif). Comparisons between two groups were done with the Student *t* test for unpaired variables. Comparisons between more than two groups were analyzed by ANOVA. Data are reported as mean ± SD. *P* values <0.05 were considered as statistically significant.

## Results

### Effect of 6-MP on airway epithelial cell viability

**Fig. 1:**
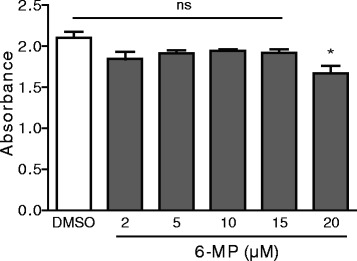
Effect of 6-MP on airway epithelial cell viability. Serum-starved NCI-H292 cells were pre-treated with 6-MP at the indicated concentrations and MTT assays were performed to assess cell proliferation. Values represent mean ± S.D. *, *p* ≤ 0.05; ns = non-significant

6-MP is an immunosuppressive drug and is known to associate with inhibition of proliferation of cells such as T-lymphocytes, smooth muscle cells, endothelial cells and intestinal epithelial cells, we sought to investigate the effect of 6-MP on viability of airway epithelial cells [19, 27–30]. To study this, a MTT assay was performed using various concentrations of 6-MP in mucoepidermoid carcinoma NCI-H292 cells. We found that 6-MP has no effect on cell proliferation at concentrations up to 15 μM, however it inhibits cell proliferation at a concentration of 20 μM (Fig. 1). No cell cytotoxicity was observed at concentrations up to 15 μM (data not shown). Therefore, we chose to study the effect of 6-MP at 10 μM in the following experiments as it was also shown to be effective in our previous studies with gut epithelial cells [19, 29].

### Inhibition of the inflammatory response of airway epithelial cells by 6-MP

**Fig. 2:**
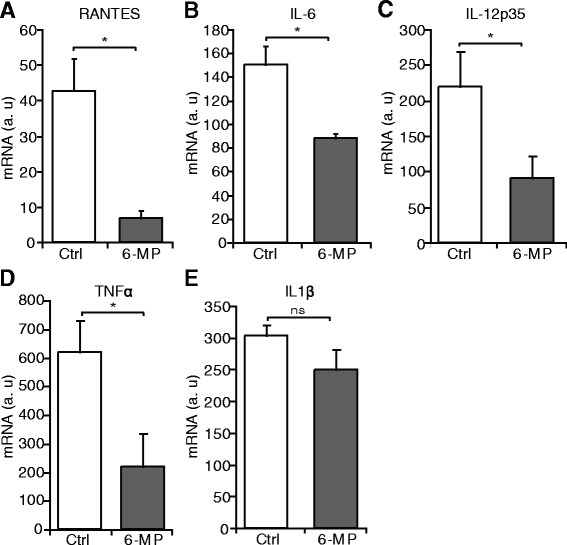
6-MP decreases the inflammatory response in airway epithelial cells. Serum-starved NCI-H292 cells were pre-treated with 6-MP and then stimulated with TNFα for 6 h. RT-PCR was performed to assess mRNA expression of RANTES (**a**), IL-6 (**b**), IL-12p35 (**c**), TNFα (**d**), and IL-1β (**e**). Values represent mean ± S.D. *, *p* ≤ 0.05. a.u = arbitrary units

We and others previously demonstrated that 6-MP decreases the inflammatory response in various cells such as endothelial cells, smooth muscle cells and gut epithelial cells [19, 29, 30]. As inflammation is also a key event in airway diseases, we investigated the effect of 6-MP on inflammation in NCI-H292 cells. 6-MP significantly decreased TNFα-induced mRNA expression of several proinflammatory cytokines such as RANTES, IL-6, IL-12, and TNFα, but not IL-1β (Fig. 2). In addition, 6-MP decreases PMA-induced mRNA expression of cytokines in NCI-H292 cells (Additional file 1: Figure S1E-F). Similar data were obtained in mouse alveolar epithelial MLE-12 cells (Additional file 1: Figure S1A-B). Altogether, these data indicate that 6-MP has an anti-inflammatory function in airway epithelial cells.

### 6-MP inhibits activation of the NFκB pathway

**Fig. 3:**
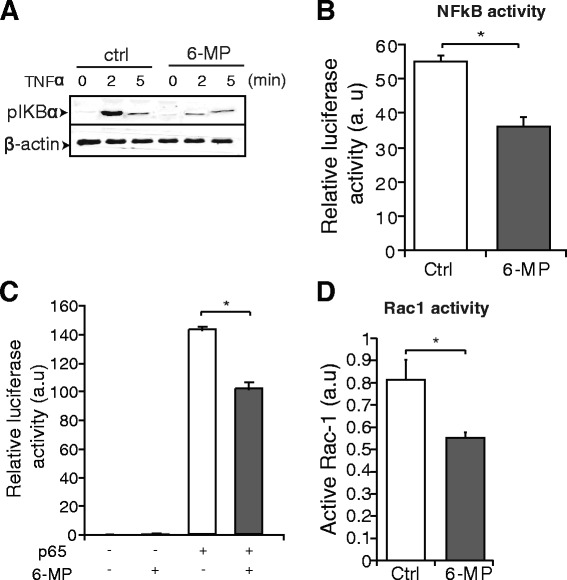
6-MP inhibits activation of the NFκB pathway. **a** Serum-starved NCI-H292 cells were pre-treated with 6-MP and then stimulated with TNFα for the indicated time periods. Western blot analysis for pIkBα was performed and β-actin was used as a loading control. **b** NCI-H292 cells were transfected with a NFκB-reporter plasmid and TNFα-induced luciferase activity was measured in the absence and in the presence of 6-MP. **c** The NFκB subunit p65 was overexpressed together with the NFκB-reporter plasmid and luciferase activity was measured after 36 h. The transfection efficiencies were normalized using Renilla luciferase co-transfection. **d** Rac1 activity is decreased by 6-MP in MLE-12 cells. Values represent mean ± S.D. *, *p* ≤ 0.05. a.u = arbitrary units

NFκB is a pleiotropic transcription factor that is activated in response to inflammatory cytokines, mitogens, and infections in airway epithelial cells [11]. Having established that 6-MP inhibits activation of the NFκB pathway in endothelial cells [29], and based on its profound inhibitory effect on inflammatory response in NCI-H292 cells, we hypothesized that 6-MP inhibits the NFκB pathway in NCI-H292 cells. NCI-H292 cells were serum-starved for 24 h and pretreated with 6-MP followed by stimulation with TNFα for the indicated time points. Western blot analysis shows that 6-MP inhibits TNFα-induced phosphorylation of IκBα, an inhibitory unit of NFκB (Fig. 3a). To corroborate these findings, we performed a luciferase assay using an NFκB luciferase reporter plasmid. Consistent with the above findings, 6-MP significantly reduced TNFα-induced NFκB activity in NCI-H292 cells (Fig. 3b). Previous studies showed that 6-MP exhibits an anti-inflammatory function through inhibition of the NFκB subunit p65 in a rat model of subarachnoid hemorrhage [31]. Therefore, we investigated the effect of 6-MP on cells overexpressing the NFκB subunit p65. We found that 6-MP attenuates p65 activity indicating that 6-MP directly affects the transcriptional activity of NFκB (Fig. 3c). In addition, 6-MP decreases PMA-induced NFκB activity in MLE-12 cells (Additional file 1: Figure S1C). In endothelial cells and gut epithelial cells, 6-MP inhibits Rac1 activity [19, 29]. As a measure of Rac1 activity GTP-bound Rac1 was measured in MLE-12 cells and show to be reduced by 6-MP (Fig. 3d). Taken together, these data demonstrate that 6-MP reduces the inflammatory response through inhibition of the NFκB pathway at different levels.

### 6-MP attenuates Muc5ac mucin gene expression

**Fig. 4:**
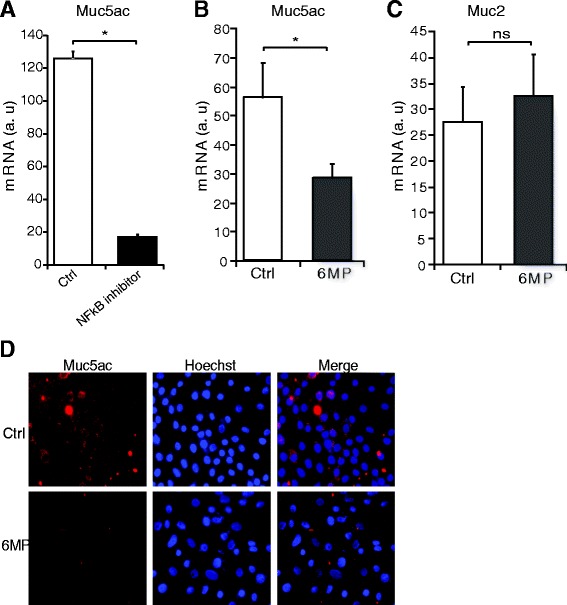
6-MP decreases Muc5ac mucin gene expression. **a** Serum-starved NCI-H292 cells were treated with a NFκB inhibitor and then stimulated with TNFα. RT-PCR was performed to assess mRNA expression of Muc5ac. **b-c** Serum-starved NCI-H292 cells were pre-treated with 6-MP and then stimulated with TNFα, and RT-PCR was performed to assess mRNA expression of Muc5ac (**b**) and Muc2 (**c**). Values represent mean ± S.D. *, *p* ≤ 0.05. **d** Muc5ac protein expression was determined by immunofluorescence using the appropriate antibody, and Hoechst was used for nuclear staining. a.u = arbitrary units

Numerous studies reported that the NFκB pathway is involved in regulation of Muc5ac gene expression in airway epithelial cells [11]. Indeed, we also found that an NFκB inhibitor markedly decreased mRNA expression of TNFα-induced Muc5ac mucin gene expression in NCI-H292 cells, confirming the previously published results (Fig. 4a). Since 6-MP reduces activation of the NFκB pathway, we hypothesized that 6-MP may regulate Muc5ac gene expression. To test our hypothesis, we performed RTPCR analyses for Muc5ac gene expression following treatment with 6-MP. As expected, we found that 6-MP significantly decreased TNFα-and PMA-induced Muc5ac gene expression (Fig. 4b; Additional file 1: Figure S1D). In addition to Muc5ac, Muc2 is also associated with inflammatory airway diseases such as chronic bronchitis, and cystic fibrosis [32, 33]. We therefore analyzed the mRNA expression of Muc2, but 6-MP has no effect on the mRNA expression of Muc2, suggesting the selective regulation of Muc5ac by 6-MP (Fig. 4c). We next investigated the effect of 6-MP on Muc5ac protein expression using an immunofluorescent assay. Consistent with mRNA data of Muc5ac, 6-MP strongly inhibits the TNFα-induced Muc5ac protein expression (Fig. 4d). Altogether, we conclude from these experiments that 6-MP-mediated inhibition of NFκB reduces Muc5ac gene expression.

### Mucus production is decreased by 6-MP

**Fig. 5:**
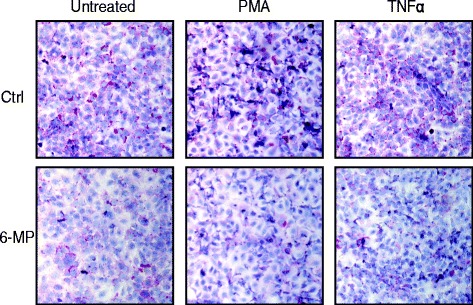
6-MP inhibits mucus production in airway epithelial cells. Serum-starved NCI-H292 cells were treated with 6-MP and then stimulated with PMA or TNFα. Mucus glycoconjugates were visualized by PAS staining. Hematoxylin staining was incorporated as a counterstain to visualize the nuclei

To further substantiate above findings, we performed PAS staining to test whether 6-MP has any effect on overall mucus production. Serum-starved NCI-H292 cells were pretreated with 6-MP followed by stimulation with PMA or TNFα. Similar to TNFα, PMA has also been shown to induce mucus production in airway epithelial cells [34]. In line with reduced Muc5ac gene expression, 6-MP markedly attenuated mucus production in the untreated, PMA- and TNFα-stimulated NCI-H292 cells (Fig. 5).

## Discussion

Excessive mucus production is an important hallmark of airway diseases such as asthma, cystic fibrosis, and chronic obstructive pulmonary disease [8]. A promising approach to attenuate airway inflammation is identification and development of a useful drug that inhibits secretion and production of mucins, the major constituents of airway mucus. 6-MP is an immunosuppressive drug and was reported to exhibit various biological effects such as anti-inflammatory and immunomodulatory functions [19, 20, 29]. Although 6-MP has been used to treat asthmatic patients, its function as well as the mechanism responsible for its action on airway epithelial cells is unknown. In this study, we investigated the potential effect of 6-MP on mucin gene expression and mucus production in airway epithelial cells. For these studies, we chose human NCI-H292 mucoepidermoid carcinoma and mouse lung epithelial MLE-12 cells, widely used model systems to study mucin production, because various inflammatory stimuli induce mucin gene transcription in these cells [35]. We demonstrated, to the best of our knowledge for the first time, that 6-MP significantly reduces both TNFα- and PMA-induced Muc5ac mucin gene expression and mucus production through inhibition of airway inflammation mediated by NFκB pathway.

As an immunosuppressive drug, 6-MP has been shown inhibit proliferation of different cells such as lymphocytes, smooth muscle cells, endothelial cells and intestinal epithelial cells [19, 27–30]. Our present data demonstrate that 6-MP has no effect on the proliferation of lung airway cells, however 6-MP exhibits cytotoxic effects at concentrations above 15 μM. Intestinal epithelial cells are more sensitive to 6-MP, as a 10 μM concentration was shown to inhibit the proliferation of these cells [19], which may require further research.

Allergic asthma is well characterized by mucus hypersecretion and airway inflammation which eventually leads to airway obstruction [5–7]. Airway epithelial cells, in addition to other cells such as dendritic cells, airway smooth muscle cells and lymphocytes, contribute to airway inflammation in asthma involving enhanced activation of the NFκB pathway. NFκB is a crucial regulator of inflammation and immunity, and is activated in bronchiolar epithelium both in humans and mice [12–14]. Inhibition of NFκB activity has been associated with a strong down-regulation of many of the molecular events that culminate in airway inflammation and structural damage of the lung in asthma. 6-MP has been demonstrated to be effective in the treatment of inflammatory diseases such as inflammatory bowel disease, rheumatoid arthritis and asthma, probably through modulation of the NFκB pathway [17, 29, 31]. Our present data further extend these findings showing that 6-MP attenuates expression of several proinflammatory cytokines, which are down-stream targets of NFκB, in airway epithelial cells. We demonstrate that 6-MP decreases Rac1 activation, similarly as in other cell types, however, in lung epithelial cells this did not result in apoptosis as was observed in T cells [20]. Given that Rac1 is an inducer of IκBα phosphorylation, 6-MP inhibits this phosphorylation resulting in reduced NFκB activity in airway epithelial cells. The mere fact that overexpressed p65 as well as PMA-induced NFκB activity is lowered may indicate that 6-MP also has a more direct inhibitory effect on NFκB, for which at present the exact underlying mechanism is unknown.

Airway epithelial cells produce mucins, a class of mucus glycoproteins, that are crucial in maintaining epithelium homeostasis [3]. Under diseased conditions such as asthma, exaggerated airway epithelial mucin production leads to mucous plugging and ultimately to death [8]. Although other mucins are present, Muc5ac is a major constituent of airway mucus in humans [9, 10]. It is well documented that multiple inflammatory stimuli such as TNFα induce the expression of Muc5ac through activation of NFκB in airway epithelial cells [11]. We have shown here that 6-MP attenuates TNFα-induced Muc5ac gene expression and mucus production in NCI-H292 cells. Even though Muc2, like Muc5ac, contains an NFκB response element in its promoter [36], 6-MP failed to suppress Muc2 gene transcription. Apparently, the signaling pathways to induce Muc2 expression are different from those of Muc5ac, also demonstrating the selective regulation of 6-MP on Muc5ac. To gain insight on the effect of 6-MP on overall mucus production, PAS stainings were performed, revealing a dramatic decrease. 6-MP strongly inhibits both TNFα- and PMA-induced mucus production probably through inhibition of NFκB pathway. Although we did not investigate the expression of all known mucins, one may suggest that, in addition to Muc5ac, there are more 6-MP sensitive mucin genes.

## Conclusions

In summary, we demonstrated that the immunosuppressive drug 6-MP inhibits inflammatory response induced by TNFα in human NCI-H292 and mouse MLE-12 lung cells. In addition, 6-MP attenuates TNFα-induced Muc5ac expression and total mucus production through inhibition of activation status of Rac1 and IκBα phosphorylation resulting in reduced NFκB activation in NCI-H292 cells. The data presented in the current study disclosed a previously unknown role of 6-MP in airway epithelial cells as an efficacious mucoregulator. As these experiments were performed in cultured cells, results may not be generalizable to the airway epithelium *in vivo* and future studies should focus on testing of 6-MP in animal models of allergic airway inflammation.

## Additional file

Additional file 1: Figure S1. 6-MP decreases PMA-induced inflammatory response in airway epithelial cells. **A-B;** Serum-starved MLE-12 cells were pre-treated with 6-MP and then stimulated with PMA (1 nM) for 6 h. RT-PCR was performed to assess mRNA expression of CXCL1 (A) and RANTES (B). **C;** MLE-12 cells were transfected with an NFκB-reporter plasmid and PMA-induced luciferase activity was measured in the in the presence of 6-MP. **D-F;** Serum-starved NCI-H292 cells were pre-treated with 6-MP and then stimulated with PMA (1 nM) for 6 h. RT-PCR was performed to assess mRNA expression of Muc5ac (D), IL-1β (E), and RANTES (F). Values represent mean ± S.D; *, *p* ≤ 0.05; a.u = arbitrary units.

## Abbreviations

6-MP: 6-Mercaptopurine
FCS: Fetal calf serum
NFκB: Nuclear factor kappa-light-chain-enhancer of activated B cells
PAS: Periodic Acid Schiff
TNF: Tumor necrosis factor
